# Quality of Life in Older Adults with Benign Prostatic Hyperplasia

**DOI:** 10.3390/healthcare8020158

**Published:** 2020-06-04

**Authors:** Sewon Park, Jeong-min Ryu, Munjae Lee

**Affiliations:** Department of Medical Device Management and Research, SAIHST, Sungkyunkwan University, Seoul 06355, Korea; se10919@g.skku.edu (S.P.); jungmin94@g.skku.edu (J.-m.R.)

**Keywords:** health-related quality of life (HRQOL), Benign Prostatic Hyperplasia (BPH), older adults, subjective health status, physical activity

## Abstract

The purpose of this study is to identify factors that affect health-related quality of life (HRQOL) of older patients with Benign Prostatic Hyperplasia (BPH) and suggest ways to improve the same. Through this, we will improve the self-management practice of patients and promote the treatment of BPH in older patients. The 2015 Korea Health Panel Survey data were used in this study. A total of 422 BPH patients aged 65 or older were included. Logistic regression analysis was conducted to identify factors affecting the HRQOL of older patients with BPH. General characteristics of factors affecting older patients with BPH included income level and type of insurance. In addition, among medical-related characteristics and health behavior factors, subjective health status, unmet medical care needs, moderate physical activity, sitting time, and drinking influenced the HRQOL. Therefore, in order to improve the HRQOL of adult patients with BPH, it is necessary to improve medical accessibility by strengthening primary care. In addition, it is necessary to increase the amount of activity in daily life through healthcare medical devices.

## 1. Introduction

As the average life span has recently increased, so has interest in health policies, the desire to live without disease and disability, and efforts to improve the health-related quality of life (HRQOL) of patients. In particular, the importance of healthcare for the middle-aged, as well as health problems affecting the aging population are being emphasized [1,2]. Since medical expenditures are increasing as the aging generation expands rapidly, policies are required to concentrate efforts for them [3,4]. With age, patients are increasingly developing chronic diseases; Benign Prostatic Hyperplasia (BPH) is a representative disease that appears in males during the aging process. The deterioration of the lower urinary tract is known as a common symptom of senile disease in males. In particular, BPH accounts for almost 80% of the diseases that cause lower urinary tract symptoms, and is commonly experienced by 25% of males worldwide. BPH, which usually occurs in men aged in their 40s and 50s, induces symptoms when urinating, such as tension, incomplete bladder emptying, miction pain, etc., and acts as a factor in degrading the HRQOL. In addition, unless treated, it could cause cystoliths and renal insufficiency; therefore, continuous care is necessary [5,6,7].

BPH is a disease that applies restrictions to daily life and physically acts as a cause of urinary tract infection and odor, making it one of the causes that degrades a patient’s subjective health status and HRQOL [8]. HRQOL refers to the subjective well-being status each individual may perceive in terms of physical, mental, socioeconomic, and spiritual aspects [9]. BPH affects HRQOL by inducing psychological stress in patients due to anxiety and deterioration of their social function. The more severe the symptoms of BPH, the lower the HRQOL becomes; HRQOL of patients with BPH who experience worsening symptoms due to aging has been shown to be significantly reduced [10,11,12]. Particularly, as HRQOL acts as a major factor causing behaviors to manage diseases, improving the HRQOL can induce the treatment pursuing behavior of patients with BPH. Furthermore, BPH is usually treated with medication and surgery; however, effective treatment results can be expected only when an individual’s health behavior improves simultaneously [13,14,15]. Therefore, through improvement in the HRQOL of patients with BPH, self-care of patients may increase.

The elderly experience difficulties in health care due to declined physical activity, economic status, psychological problems, etc.; in patients with BPH who need self-care, the older they become, the lower their knowledge acquisition, cognitive abilities, etc. in disease management, as compared to younger adults. Hence, in this study, we intend to seek solutions to improve the health-related QOL of older patients with BPH by identifying factors affecting the same. Through this, we will suggest measures to promote medical treatment in older patients with BPH by improving the execution power for their self-care.

## 2. Methods

### 2.1. Data Source

This study used the 2015 data from the yearly data of the Korea Health Panel Survey (KHPS) 2008–2015. The KHPS is a government-approved statistical survey (no. 92012) where the Korea Institute for Health and Social Affairs and the National Health Insurance Corporation organize a consortium to yield baseline data, including the use of health and medical care services, expenditure levels, and health behaviors. The materials prepared through the nationwide project of the KHPS focus on the representative statistical calculations of the people’s current state of health and medical care, medical expenses, and finances in the national and municipal units. The 2015 KHPS, the 10th survey, was conducted from March to September 2015, targeting 15,263 people from 5284 households for investigation.

BPH is known to frequently occur in males [16]. In this study, only men who visited medical institutions due to BPH were used, and the data sources were utilized as follows. First, it was used to determine the prevalence of patients with BPH. A patient with BPH was defined as those who responded that the doctor diagnosed BPH. Diseases of the urinary system investigated by the KHPS include Urinary tract stones (N20–N23), Kidney failure (N17–N19), and Cystitis (N30). Among them, only patients diagnosed by BPH (N40) were extracted. Second, patients diagnosed with BPH and undergoing treatment were extracted. The KHPS provides information on medical use, especially diagnosis, treatment, and continuous treatment. In this study, patients who were diagnosed with BPH and responded to treatment with medication and surgery were extracted. In the case of drug treatment, patients who responded to regular visits to medical institution visit types were extracted and targeted for patients who are regularly treated for BPH.

The KHPS is focused on tracking causal relationships between medical utilization behavior, health behavior as a database that can identify factors that directly or indirectly affect medical care services. Although information such as demographic characteristics of patients, income, diagnostic and treatment, use of medical services, and unmet medical care can be grasped, there is a limit to grasping the severity of the disease. In this study, among the patients with BPH, patients who are being treated with BPH to extract using the items of treatment or examination provided by the KHPS. Therefore, a total of 543 men who were diagnosed by a doctor with BPH and who received regular outpatient treatment and surgical treatment were extracted. Moreover, 18 people who did not respond to the questions associated with health behavior and medical care-related characteristics were excluded from the extracted patients. In addition, since this study analyzes the factors affecting the quality of life of patients with BPH over 65,103 patients with BPH under 65 were excluded. The final study subjects utilized through this process were 422 patients with BPH over 65 years of age.

**Figure 1:**
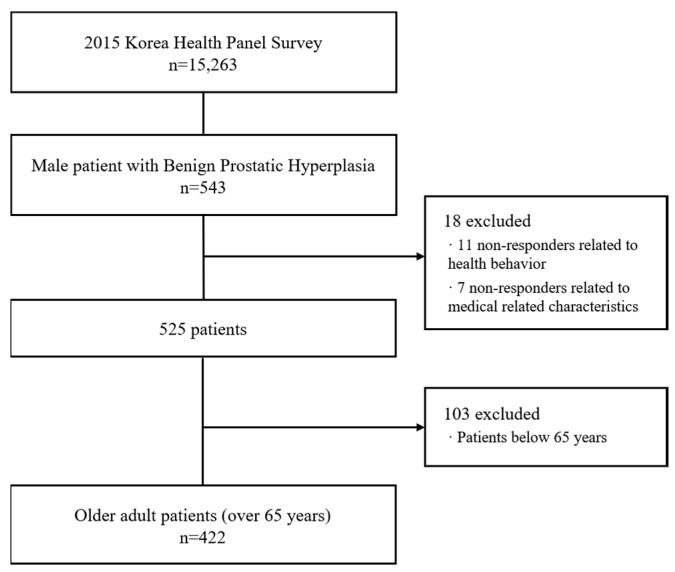
Flowchart.

The KHPS is approved by the ethical committee of the Korea Institute for Health and Social Affairs and the National Health Insurance Service. The requirement for informed consent was waived because data in the KHPS are anonymized in adherence to strict confidentiality guidelines (Figure 1).

### 2.2. Description of Variables

#### 2.2.1. General Characteristics of Subjects

The variables used in this study were as follows: age, education level, income, economic activities, and forms of medical security. The participants were classified according to age, with an age of 65 years as the cutoff. In terms of education level, the participants were grouped into those who graduated high school and those who did not graduate high school. In terms of income, the participants were divided into groups based on the annual gross household income: those who earned more than 3 million won and those who earned less than 3 million won. Frequent urination and residual urine in patients with BPH have negative physical and psychological effects on them, which may lead to a decrease in economic activity and sometimes result in personal economic losses [17]. Hence, to examine how the HRQOL could be affected depending on the economic activities, they were divided into economic-life active and inactive individuals using the following item to determine whether they were engaged in economic activities: “Are you working for your income?” Since the medical costs related to the treatment of BPH tends to increase over time, analyzing the HRQOL according to the type of insurance is necessary. Therefore, type of insurances were classified into health insurance subscribers and medical beneficiaries [18].

#### 2.2.2. Characteristics of Health Behavior and Medical Related Characteristics of Research Subjects

This study aims to induce treatment pursuit by improving the quality of life to change the health behavior of patients with BPH. The KHPS includes socioeconomic factors, health level, and health behavior for determinants of medical use. Since the subject of the study was diagnosed with BPH, the focus was on the factors of BPH patients’ medical use, health behavior, and additional possession of diseases such as chronic diseases, rather than the factors causing BPH. In the case of health behaviors, smoking, physical activity, and drinking were included to ensure that patients with BPH are basically managed for health. Therefore, subjective health status, unmet medical care needs, chronic disease, physical activity, time spent sitting, smoking, and drinking were used as variables associated with the health behaviors and medical-related characteristics of participants. Regarding subjective health status, the questionnaire items in the KHPS were utilized, which were rated on a five-point scale. The higher the score, the higher the negative effects on health; subjective health status was divided into “good” and “bad.” Unmet medical care needs were divided into presence and absence of unmet medical care needs, based on the participants’ response to the following question: “In the previous year, did you have any experience that you have needed to receive treatments or examinations in clinics and hospitals but you could not?” In addition, those who had at least one chronic disease were classified as patients with chronic disease [19,20]. Since the probability of having chronic disease increases with age, the presence or absence of chronic disease was used as a variable to measure the factors affecting the quality of life according to the accompanying diseases of patients with BPH. By calculating the amount of energy used during physical activities into metabolic equivalent task (MET)-minutes according to the energy requirements of the respondents, which were determined based on their response to the survey, physical activities were classified into intense and moderate. Because the risk of BPH increases in those who sit for a long time, we included time spent sitting as a variable. The National Health Statistics indicated that the daily sitting time for males is 8 h and that for females is 7.8 h; since this study was only conducted on men, daily sitting time was divided based on the 8 h threshold [21,22]. For smoking status, patients who smoked in the past but not currently or have never smoked were classified as non-smokers, and those who smoked every day or sometimes were classified as smokers. With regard to drinking status, patients who have not drunk in the past 1 year were classified as non-drinkers, while the remaining were classified as drinkers [23,24].

#### 2.2.3. Health Related Quality of Life

Health-related QOL was calculated using the European Quality of life—Five Dimension (EQ-5D) scores in the KHPS. The items in the EQ-5D questionnaire were also included in the KHPS since 2009. The five health-related QOL items, measured in the KHPS, were motor ability, self-management, daily activities, pain/inconvenience, and anxiety/depression. Each question is rated on a three-point scale: “not troubled,” “somewhat troubled,” and “troubled” [25]. The EQ-5D only yields a single score between 0 and 1 through weights, which means that a score closer to 1 indicates a better HRQOL. In this study, to divide the participants into the deteriorated and non-deteriorated groups in terms of their health-related QOL, all the responses to the questionnaire items were re-computed and converted to 0 point for “troubled’ and 0 point for “not troubled”; we converted them to a single score of 0–1. Participants with higher scores were included in the non-deteriorated group [26,27,28].

### 2.3. Statistical Analysis

The analysis process of this study is as follows.

First, frequency analysis was performed to identify sociodemographic characteristics of older patients with BPH. Second, through logistic regression analysis, factors influencing HRQOL were derived according to general characteristics, health behavior, and medical-related properties of the research subjects. The SPSS 25.0 (SPSS Inc., Chicago, IL, USA) Program was utilized for the analysis.

## 3. Results

The general characteristics of the subjects are reported in Table 1. In terms of the level of education, 362 people (85.8%) had not graduated high school; high school graduates or higher accounted for 60 people (14.2%), indicating that most patients had a low level of education. Regarding income level, 253 people (60.0%) earned less than KRW (Korean Won) 3 million, whereas 169 people (40.0%) earned KRW 3 million or higher, suggesting that patients with low income were in the majority. Moreover, 270 patients (64.0%) were economically active, whereas 152 patients (36.0%) were not. With regards to types of insurance, 371 people (87.9%) were health insurance subscribers, and 51 people (12.1%) were medical beneficiaries; 141 patients (33.4%) perceived their subjective health status as good, but 281 patients (66.6%) considered it bad, indicating that most patients were inclined to think of their own health status as not good. In all, 79 patients (18.7%) experienced unmet medical care, whereas 343 patients (81.3%) did not, and 222 patients (52.6%) had chronic diseases, compared to 200 patients (47.4%) who did not. Furthermore, 280 patients (66.4%) participated in moderate physical activities, whereas 142 patients (33.6%) did not. On the other hand, 220 patients (52.1%) engaged in intense physical activities, while 202 patients (47.9%) did not, suggesting that more patients participated in moderate physical activities. In addition, 256 patients (60.7%) sat for 8 h or less, while 166 patients (39.35) sat for more than 8 h daily. Furthermore, 50 patients (11.8%) were smokers, whereas 372 patients (88.2%) were non-smokers, thus, a majority of patients were non-smokers. Finally, 197 patients (46.7%) consumed alcohol, compared to 225 patients (53.3%) who did not.

Logistic regression analysis was performed to identify factors affecting health-related QOL of older patients with BPH. To conduct the analysis, sociodemographic characteristics, medical use-related characteristics, and health behaviors were set as independent variables; the deteriorated group (0) and the non-deteriorated group (1) with regards to HRQOL were used as dependent variables. Among sociodemographic features, factors affecting the HRQOL of older patients with BPH included their economic activity and type of insurance. Exp(B) referred to the probability of belonging to the group with internal value 1 (non-deteriorated group), compared with the probability of belonging to the group with internal value 0 (deteriorated group) according to the increase of the variable value 1 [29,30]. Therefore, if Exp(B) is greater than 1, the higher the value of the variable, the more likely it is to belong to the deteriorated group—the group with internal value 1. In other words, for older patients with BPH, patients with economic activity experienced an improved HRQOL of 2.467 times, and that patients who have health insurance experienced an HRQOL that was improved 2.193 times. The results are presented in Table 2.

Among medical use-related features and health behavior factors affecting the HRQOL of older patients with BPH were subjective health status, unmet medical care, moderate physical activity, time spent sitting, and drinking alcohol. The HRQOL of patients who perceived their subjective health status to be bad was 0.262 times lower than those who considered their subjective health status as good; patients who had experienced unmet medical care saw a decreased HRQOL by 0.154. The HRQOL of patients involved in moderate physical activities was found to be 2.557 times higher than those who did not participate in them, suggesting that intense physical activity was not statistically significant. Thus, it can be judged that moderate physical activities, such as walking, gymnastics, yoga, etc., rather than intense physical activities such as climbing, swimming, and running, may increase HRQOL. In addition, patients who sat for long hours had a further 0.452-fold decrease in HRQOL than those who did not; similarly, patients who drank alcohol showed an HRQOL decreased by 0.521, as compared with non-drinking patients. Results of the analysis are reported in Table 3.

## 4. Discussion

In this study, we intended to seek measures to improve patients’ HRQOL and promote medical treatment by analyzing factors affecting the HRQOL of older patients with BPH. The results indicated that influencing factors included economic activity, type of insurance, subjective health status, unmet medical care, moderate physical activity, sitting time, and drinking.

The higher the economic activity of such a patient, the better their HRQOL, as evidenced in existing research findings wherein the higher the socioeconomic level—such as income, occupational position, etc.—the lower the prevalence of disease, and thus, the health level increases [31,32,33]. Therefore, elder people with income through economic activities were more likely to consult a doctor on their health status as they had significant execution power for health behaviors to improve health status, thus leading to the reduced prevalence of the disease. In addition, patients who had health insurance and those who had not experienced unmet medical care demonstrated a better HRQOL. This is consistent with research findings that older patients who experienced unmet health care saw reduced HRQOL, and the more the patient shares his medical costs, the lower his HRQOL becomes. Furthermore, consistent with research findings, patients with BPH who have been continuously treated have a better HRQOL than those who have not regularly used health care [13,27,34,35,36,37]. Considering these findings, it is necessary to reduce the burden on the use of medical services by improving accessibility to health services for older patients with BPH. In particular, since the older population is restricted from receiving continuous treatment due to limitations in physical activity, financial reasons, etc., it is deemed necessary to alleviate older patients’ need to share.

It was observed that the higher the subjective health status, the better the HRQOL of such patients. As aforementioned, patients with BPH tend to suffer from disabilities in daily life due to lower urinary tract symptoms, which may cause depression, anxiety, and stress and thus negatively affect subjective health status [38,39,40]. Symptoms of BPH, unlike other chronic diseases such as hypertension and diabetes, are often perceived as less serious. Some may simply consider it an inconvenience in daily life, while others do not think treatment is necessary and that the condition it will naturally improve [41]. We can judge that as delay occurs in the treatment of BPH for this reason, the patient’s subjective health state lowers and HRQOL consequently reduces. In particular, as people consider the condition to be a decrease in physical function due to aging, it will be necessary to create a medical environment where symptoms of BPH can be actively tested and treated. Light physical activities, such as walking, and sitting time affected the HRQOL of the older patients with BPH. This is in line with previous studies that physical activity, psychological pains (such as anxiety and stress), and sitting time are closely related to the HRQOL and that physical activities may alleviate psychological pains and sitting time is associated with psychological pains [33,42,43]. In addition, it is consistent with existing research findings that patients with BPH who were highly physically active showed lower frequency for lower urinary tract symptoms than those who were sitting extensively [17,44]. As moderate physical activity, rather than intense physical activity, had a positive effect on the HRQOL of older patients with BPH, physical activities such as walking will be required to improve HRQOL. Moreover, the shorter the sitting time, the higher the HRQOL, which could be considered as similar to studies that found that decreasing sitting time could reduce the risk of BPH [45]. That is, the physical activity of patients with BPH improves physical function and overall health and alleviates symptoms of BPH. In addition, physical activity is considered to improve the quality of life of patients with BPH, because the improvement of the patient’s physical health function improves the quality of life. This is also consistent with findings that as older adults’ participation ratio in economic activities becomes lower, their sitting time is reduced at normal times and their HRQOL also gets lower, along with lowered physical function [46]. Therefore, in terms of the older patients with BPH, reducing sitting time through increased physical activities in daily life may improve HRQOL. Consuming alcohol decreased the HRQOL of the older patients with BPH. These findings differed from preceding studies in that moderate drinking has an influence on reducing lower urinary tract symptoms and that drinking does not rapidly increase the risk of BPH [47,48,49]. However, in the older population, excessively high alcohol consumption may cause physical damage or difficulties in managing diseases, such as forgetting to take drugs. In addition, since BPH is often treated with medication, older patients with BPH must avoid excessive drinking—awareness in this regard is required.

The limitations of this study are as follows. First, further analysis is needed for patients with BPH who have chronic disease. Studies suggest that chronic disease and BPH are correlated and that increasing metabolic syndrome may cause lower urinary tract symptoms and increase the risk of BPH. Second, future studies will be able to prevent BPH via further analysis on BPH and chronic diseases in older adults. Neither chronic disease nor BPH occur due to a single cause, and because they repeat improvement and deterioration, longitudinal studies using panel data are necessary. In recent years, there has been an increase in patients with BPH in young and middle-aged adults due to westernized eating habits and decreased physical activity. Since BPH requires constant management, an increased prevalence in young and middle-aged adults can contribute to rising medical costs. Therefore, future studies should classify patients with BPH into young and middle-aged adults and older adults and then analyze factors affecting their HRQOL. This would enable the suggestion of factors influencing the HRQOL of patients with BPH in accordance with age, and suggest appropriate medical treatment.

Analysis of the influence factors of BPH with patients according to age seems to be able to improve the quality of life of patients by identifying the factors that pose a risk in treating patients and suggesting treatment measures by age. Third, the analysis of the factors affecting the quality of life according to symptoms was not conducted. Factors affecting the quality of life according to drug treatment and surgical treatment are likely to appear differently. In addition, the factors affecting the quality of life between people who have been cured or who do not have BPH and patients with BPH may appear differently. In a future study, the IPSS scores, which measures the severity of BPH, should be used to analyze factors affecting the quality of life according to the symptoms in the treatment process and to derive a method to improve the quality of life of patients with BPH. Through this, it would be possible to suggest appropriate treatments to relieve symptoms and improve the quality of life.

This study attempted to enhance the HRQOL of older patients with BPH by analyzing factors affecting the HRQOL of patients with BPH that remarkably reduce the HRQOL of the older population, and to improve the self-care ability of the elderly by improving their HRQOL. In particular, the discovery of factors related to medical expenditure, such as income levels and types of insurance, which limit early treatment of BPH patients, can be used as basic data to seek ways to support medical expenses for older patients with BPH, and the expansion of treatment support is expected to improve the quality of life for older patients with BPH. The discovery of health behavior factors that are in an organic relationship, such as physical activity, sedentary time, and subjective health status, improves physical activity, reduces sedentary time, and improves subjective health status. This suggests that this will improve the quality of life for older BPH with patients. In addition, this will be helpful in preventing disease by inducing the treatment pursuing activity of older patients with BPH and improving the health of older patients with BPH.

## 5. Conclusions

Accordingly, in order to improve the HRQOL in older patients with BPH who have low-income and to induce their act of medical treatment, it is necessary for the community to provide medical care services. This could be achieved by improving the accessibility to healthcare through strengthening primary medical care. Furthermore, expanding health insurance coverage for the checkup of BPH will help reduce medical costs for the older patients with BPH; minimally invasive treatments using robotic surgery or laser in the treatment of BPH will be able to improve the patients’ HRQOL. In the meantime, in order to improve the HRQOL of patients with BPH, it seems to be necessary to change the subjective health status into the positive state by increasing physical activity and reducing the sitting time. In particular, since moderate physical activity may improve the HRQOL, it is necessary to make older patients actively participate in physical activities by increasing healthy behaviors in daily life. Utilizing health care medical devices to help improve the lack of physical activity and bed lifestyle habits will improve the HRQOL of older patients with BPH by increasing the amount of action in daily life.

## Figures and Tables

**Table 1:** General characteristics of research participants (*n* = 422).

Characteristic	Categories	*N*	%
Education	<High school	362	85.8
	≥High school	60	14.2
Income	<300	253	60.0
	≥300	169	40.0
Economic activity	Y	270	64.0
	N	152	36.0
Type of insurance	National health insurance	371	87.9
	Assistance	51	12.1
Subjective health status	Good	141	33.4
	Poor	281	66.6
Unmet medical care	Y	79	18.7
	N	343	81.3
Chronic disease	Y	222	52.6
	N	200	47.4
Moderate physical activity	Y	280	66.4
	N	142	33.6
Intense physical activity	Y	220	52.1
	N	202	47.9
Sitting time	<8 h	256	60.7
	≥8 h	166	39.3
Smoking	Y	50	11.8
	N	372	88.2
Drinking	Y	197	46.7
	N	225	53.3

**Table 2:** Factors affecting health-related quality of life (general characteristics).

Variable (Reference)		B	S.E.	Wald	*p*	Exp(B)	95% Cl for Exp(B)	
							Lower	Upper
Education (under high school)	Over high school	0.548	0.334	2.691	0.101	1.730	0.899	3.331
Income (under KRW 3 million)	Over 3 million	−0.179	0.222	0.649	0.420	0.836	0.541	1.292
Economic activity (no)	Yes	0.903	0.241	14.064	0.000	2.467 ***	1.539	3.955
Type of insurance (assistance)	National Health Insurance	0.785	0.311	6.371	0.012	2.193 **	1.192	4.036

Notes: ** *p* < 0.05, ***p < 0.001, CI: Confidence Interval.

**Table 3:** Factors affecting health-related quality of life (medical related characteristics, health behavior).

Variable (Reference)		B	S.E.	Wald	*p*	Exp(B)	95% Cl for Exp(B)	
							Lower	Upper
Subjective health status (good)	Poor	−1.340	0.261	26.385	0.000	0.262 ***	0.157	0.437
Unmet medical care (no)	Yes	−1.872	0.317	34.857	0.000	0.154 ***	0.083	0.286
Chronic disease (no)	Yes	−0.100	0.256	0.153	0.696	0.905	0.547	1.495
Moderate physical activity (no)	Yes	0.939	0.358	6.871	0.009	2.557 **	1.267	5.158
Intense physical activity (no)	Yes	−0.124	0.340	0.133	0.715	0.883	0.453	1.721
Sitting time (under 8 h)	Over 8 h	−0.793	0.255	9.703	0.002	0.452 **	0.275	0.745
Smoking (no)	Yes	0.304	0.402	0.572	0.449	1.356	0.616	2.984
Drinking (no)	Yes	−0.652	0.259	6.345	0.012	0.521 **	0.314	0.865

Notes: ** *p* < 0.05, ***p < 0.001, CI: Confidence Interval.
